# The Effect of a Mobile Health App on Treatment Adherence and Revenue at Physical Health Clinics: Retrospective Record Review

**DOI:** 10.2196/43507

**Published:** 2023-03-28

**Authors:** Robert Topp, Jay Greenstein, Jena Etnoyer-Slaski

**Affiliations:** 1 College of Nursing The University of Toledo Toledo, OH United States; 2 Kaizo Clinical Research Institute Rockville, MD United States

**Keywords:** physical health, completion of therapy, phone app, clinic charges and payments, payment, cost, physiotherapy, physical therapy, adherence, attrition, mobile phone, reminder, mobile health, mHealth, health app, mobile app

## Abstract

**Background:**

A significant number of patients do not adhere to their prescribed course of physical therapy or discharge themselves from care. Adhering to prescribed physical therapy, including attending physical therapy clinic appointments, contributes to patients achieving the goals of therapy including reducing pain and increasing functionality. Web-based platforms have been demonstrated to be effective means for managing clinical patients with musculoskeletal pain, similar to managing them in person. Behavior change techniques introduced through digital or web-based platforms can reduce nonadherence with prescribed physical therapy and improve patient outcomes. Literature also indicates that a phone-based app provided to patients, which includes a reward-incentive gamification to complement their care, contributed to a greater number of kept appointments in a physical therapy clinic.

**Objective:**

This study aims to compare the rate of provider discharge with self-discharge and the number of clinic visits among patients attending a physical health clinic who did and did not choose to adopt a phone-based app to complement their care. A secondary purpose was to compare the revenue generated by patients attending a physical health clinic who did and did not choose to adopt a phone-based app to complement their care.

**Methods:**

A retrospective analysis of all new outpatient medical records (N=5328) from a multisite physical health practice was conducted between January 2018 and December 2019. Patients in the sample self-selected the 2018 Usual Care, the 2019 Usual Care, or the 2019 Kanvas App groups. Kanvas is a customized private practice app, designed for patient engagement with their specific health care provider. This app included a gamification system that provided rewards to the patient for attending their scheduled clinic appointments. According to their medical record, each patient was classified as completing their prescribed therapy (provider discharged) or not completing their prescribed therapy (self-discharged). Additionally, the total number of clinic visits each patient attended, the total charges for services, and the total payments received by the clinic per patient were extracted from each patient’s medical record.

**Results:**

Patients in the 2019 Kanvas App Group exhibited a higher rate of provider discharge compared to patients who did not adopt the app. This greater rate of provider discharges among the patients who adopted the Kanvas app likely contributed to this group attending more clinic visits (13.21, SD 12.09) than the other study groups who did not download the app (10.72, SD 9.80 to 11.35, SD 11.10). This greater number of clinic visits in turn contributed to the patients who adopted the app generating more clinic charges and payments.

**Conclusions:**

Future investigators need to employ more rigorous methods to confirm these findings, and clinicians need to weigh the anticipated benefits against the cost and staff involvement in managing the Kanvas app.

## Introduction

### Background

Over 50 million adults (21.8% of the population) in the United States exhibit some form of a disability, while in 2010, the most prevalent disabilities resulted in limitations in mobility. The most common causes of disability were arthritis or rheumatism and back or spine problems [1]. Physical therapy aims to reduce disability and pain and improve functioning, resulting in improving the patients’ quality of life [2]. Adhering to prescribed physical therapy, including attending physical therapy clinic appointments, contributes to patients achieving the goals of therapy including reduced pain [3,4] and improved functioning [5,6]. Literature indicates that patients commonly do not adhere to their prescribed course of physical therapy. Previous investigators estimate that between 14% and 70% of patients who have been prescribed physical therapy do not complete their prescribed course of therapy or discharge themselves from care [7,8].

These findings indicate that several different factors contribute to whether a patient adheres to a prescribed course of physical therapy. These factors may either be patient oriented or related to the procedures within the clinic where the therapy is prescribed. Jack et al [8] commented that early research in this area focused on how patient-oriented factors, including low self-efficacy, depression, anxiety, helplessness, poor social support, and greater perceived number of barriers to exercise, contributed to not adhering to a prescribed course of physical therapy. Although related to adhering to prescribed physical therapy, these patient-oriented factors may be challenging to address during a physical therapy clinic visit. Other authors reported that modifying procedures within the clinic along with a personalized approach to physical therapy (Coach2Move) by a physical therapist, including providing more feedback and taking into account individuals' contextual factors, improved adherence to prescribed physical therapy [9]. An early review of the related literature examining clinical procedures concluded that prescribed physical therapy that included cognitive-behavioral change components can improve attendance at physical therapy clinic sessions [10]. After reviewing 10 RCTs, Hajihasani et al [11] concluded that cognitive-behavior change interventions, when added to routine physical therapy, were more effective than physical therapy alone in treating pain and disability and improving functional capacity variables. Recent systematic reviews and meta-analyses concluded that cognitive behavior change techniques oriented to the specific patient, including graded tasks, goal setting, self‐monitoring, problem-solving, and feedback, significantly enhanced adherence to prescribed physical therapy for chronic musculoskeletal conditions [12,13]. Thus, cognitive behavior change techniques incorporated with physical therapy appear to increase adherence with a prescribe course of physical therapy.

One approach to administering cognitive behavior change techniques designed to increase adherence with prescribed physical therapy is through a mobile digital platform or a phone-based app. In a recent study, the authors compared adherence with prescribed clinic appointments among patients attending a physical health clinic who did and did not choose to adopt a phone-based app to complement their care [14]. This app employed the cognitive behavior change techniques of reward-incentive gamification for encouraging adherence to prescribed clinic appointments. The investigators reported that the group who adopted the phone-based app had a greater (*P*<.05) number of kept clinic appointments (7.79, SD 0.25) compared to the Usual Care Group (4.58, SD 0.18). Other researchers reported that patients with musculoskeletal conditions exhibited greater adherence to their home exercise programs when the programs were provided on an app with remote support compared to paper handouts [15]. In a review of 11 clinical trials evaluating rehabilitation programs administered online or digitally, the authors concluded that these approaches to administering a rehabilitation program can improve adherence to prescribe plans of care [16]. A similar systematic review and meta-analysis assessed the effectiveness of web-based cognitive behavior change techniques (e-BMT) in the management of patients with chronic musculoskeletal pain [17]. These authors reported that cognitive behavior change techniques administered through a web-based platform is an effective means for managing patients with musculoskeletal pain similar to managing them in person. Thus, directing prescribed physical therapy through web-based or digital platforms appears to be an effective medium by which to administer cognitive behavioral interventions aimed at facilitating adherence with prescribed physical therapy. A limited number of studies have examined whether a phone-based app designed to complement a patient’s physical therapy treatment can affect the rates of provider discharge versus self-discharge. Moreover, no study has compared the revenue generated by patients attending a physical health clinic who did and did not choose to adopt a phone-based app to complement their care. The results of this study will indicate the potential of a phone-based app that complements prescribed physical therapy to impact the completion of prescribed therapy and to generated revenue for the clinic.

### Objective

The purpose of this study was to compare the rate of provider discharge with self-discharge and the number of clinic visits among patients attending a physical health clinic who did and did not choose to adopt a phone-based app to complement their care. A secondary purpose was to compare the revenue generated by patients attending a physical health clinic who did and did not choose to adopt a phone-based app to complement their care.

## Methods

### Design

A retrospective analysis of all new outpatient medical records from a multisite physical health practice was evaluated between January 2018 to December 2019. New patients admitted to this physical health practice during 2018 were assigned to the 2018 Usual Care Group. Beginning in January 2019, all new patients admitted to this practice during their initial visit were offered the opportunity to download a phone-based app, Kanvas, to complement their care. The new patients who downloaded and registered on the phone-based app self-selected the 2019 Kanvas App Group. Patients who chose not to download and register on the app self-selected the 2019 Usual Care Group. All eligible patients included in the study during 2018 and 2019 had their medical record accessed to determine if they prematurely terminated treatment against the advice of the provider (self-discharged) or if they completed their prescribed treatment (provider discharged regardless of the duration of prescribed care). The number of clinic visits, the total charges for services, and the total payments received were also extracted from each patient’s medical record. This resulted in a quasi-experimental 3-group design in which the medical records of all eligible patients initially presenting for treatment between January 2018 to December 2019 were reviewed and included in the analysis.

### Sample

The medical records of new patients who were scheduled for care during 2018 and 2019 at 5 community-based physical health clinics in the greater Washington DC area (N=5844) were initially screened to be included in this study. These clinics specialize in treating pain and increasing functional ability. Of the 5844 patients, 516 (8.8%) were excluded from the analysis because they did not attend their initial clinic appointment, they were referred to another clinic for care, they were employed by one of the targeted clinics, they died prior to completing therapy, or their clinic appointment was for a single-clinic visit (eg, clinical evaluation, massage, etc). This resulted in a total of 5328 patients being involved in the analysis, including 2523 (47%) in the 2018 Usual Care Group, 2006 (37.7%) in the 2019 Usual Care Group, and 799 (15%) self-selecting the 2019 Kanvas App Group. This sample size, employing the 2x3 cross tabulation to calculate a chi-square statistic with type 1 error set at .05 and maintaining statistical power at .8 (1-β) would be able to detect a small effect size *d*=0.05 in the different rates of self-discharge versus provider discharge among the 3 study groups.

During their initial visit, patients seeking care at the clinics in 2019 were informed they could download a free mobile app to their phone, which they could use to compliment the care they were receiving in the clinic. At this time, all patients were told about the components of the app and the reward structure as a result of using the app. The patients were also told the use of the app was voluntary and would in no way affect their care or relationship with their provider or the clinical agency.

### Ethical Considerations

This record review study was approved by the Sport & Spine Rehab Clinical Research Foundation (IRB #SSR.2021.1), which included waivers for informed consent and Health Insurance Portability and Accountability Act requirements. All data extracted from the electronic medical were deidentified, compiled without patient identifiers, and kept secured and confidential. No compensation was provided for any participants involved in the study.

### Procedure

During the initial visit at one of the targeted clinics, each patient completed an initial assessment with a practitioner (Doctor of Chiropractic) who prescribed a plan of care, which included home exercises and a series of follow-up clinic visits. During 2019, these practitioners were not blind to the patient’s decision to download and register on the phone-based Kanvas app. The plan of care prescribed by the practitioner, including the number and frequency of the follow-up clinic visits, was customized to the type and severity of the patient’s condition. The number of treatment sessions was initially determined by the provider, and based upon the patient’s clinical progress, may have been reduced or extended during the course of their therapy. When the practitioner prescribed a plan of care, the patients were informed that their account would be charged US $25 if they did not attend future scheduled visits (“no-show”) or did not contact the clinic to cancel the appointment within 24 hours of the appointment (“late cancel”).

**Figure 1:**
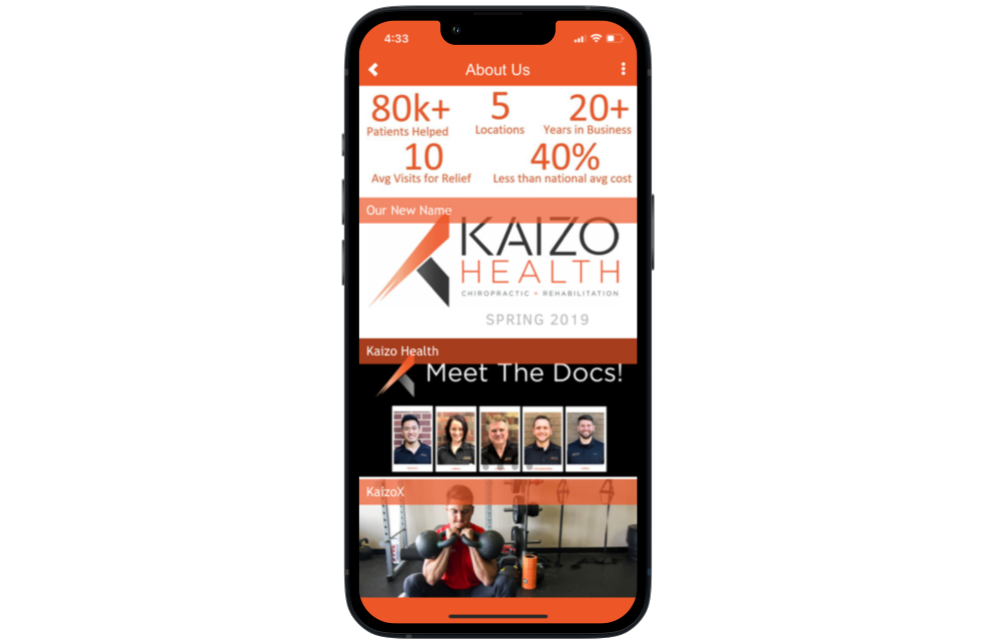
Tiles from the Kanvas app.

**Figure 2:**
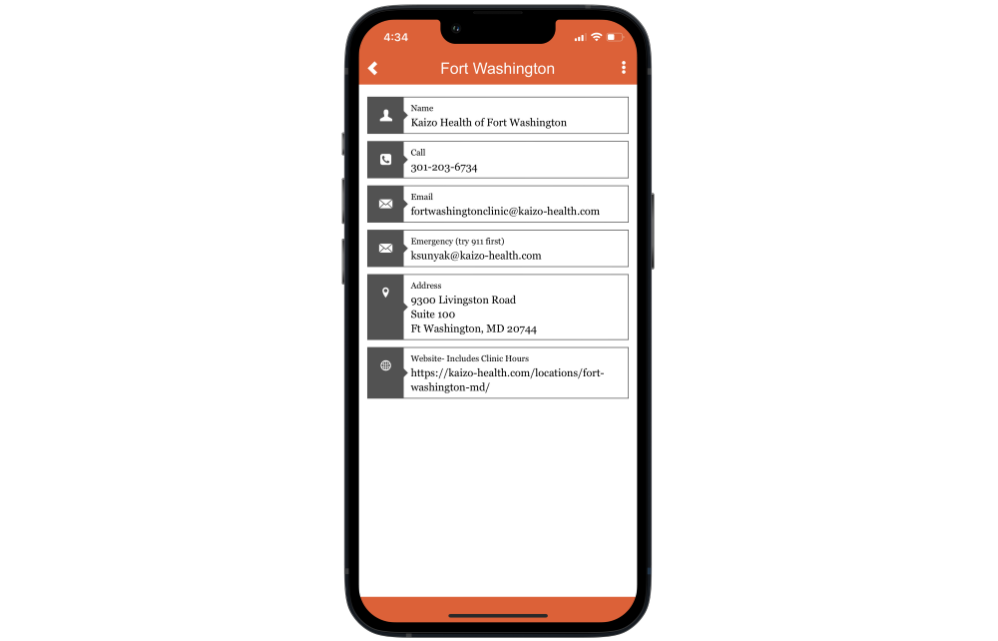
Additional tiles from the Kanvas app.

**Figure 3:**
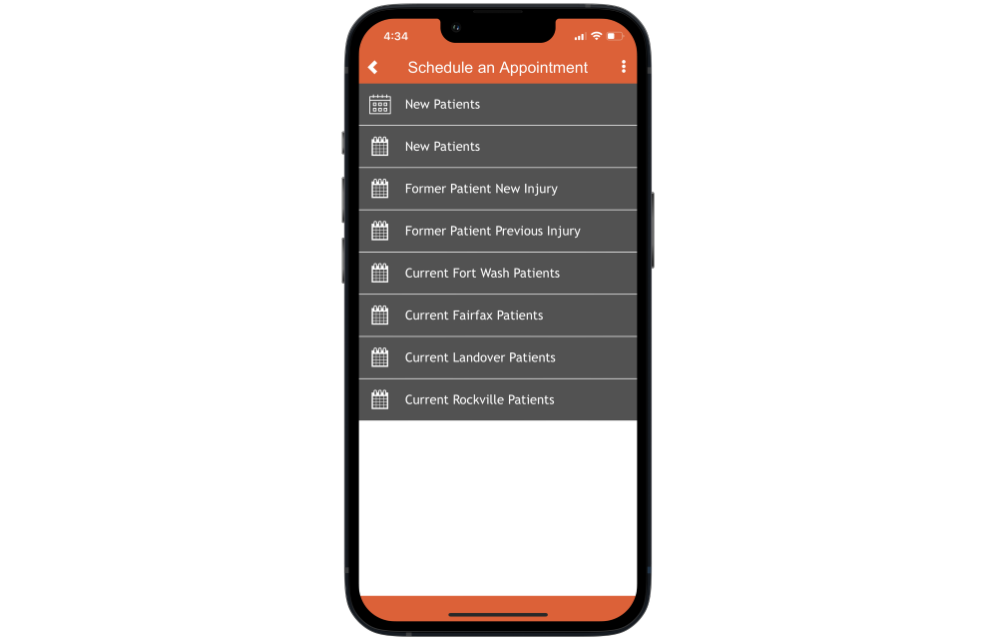
Rewards tile from the Kanvas app.

The Kanvas app is a customized private practice app, designed for patient engagement with their specific clinic. The initial screen includes various tiles in which the patient can engage with the office. These tiles include “contact us,” “about us,” “refer a friend,” “request an appointment,” “review us,” and “home exercise” (Figures 1 and 2). The app did not provide direct messaging between the patient and the provider. Additionally, the app included the cognitive behavior change technique of a built-in gamification system in the “rewards tile” (Figure 3). This feature was designed to reward the patient for attending their scheduled clinic appointments. This feature is compliant with the Office of the Inspector General, offering an item as a reward that is valued at less than US $15 once the patient completed 12 prescribed visits or were provider discharged. This feature documented a running total of the number of clinic visits the patient had attended. The feature is patient directed, in which they scan a QR code at the front desk of the clinic at every visit. When the patients reach 12 prescribed visits or are provider discharged, they are eligible for a reward.

### Outcome Variables

The medical records of all eligible patients who were initially seen in the targeted clinics over the 24-month duration of the study were reviewed during the 4-month period after their initial assessment. Based on the discharge summary documentation on the patients’ medical record, patients were classified as completing prescribed therapy and being discharged by their provider (provider discharged) or not completing their prescribed therapy and discharging themselves (self-discharged). Moreover, the total number of clinic visits each patient attended, the total charges for services, and the total payments received by the clinic per patient were extracted from each patient’s electronic medical record. Revenue generation was examined as a secondary outcome in this study. When considering the purchase of a new technology, both the return on investment and the clinical impact of the technology need to be evaluated.

### Analysis Plan

Data were extracted from the medical records of all patients identified to be eligible for the study and transcribed into an Excel (Microsoft Corporation) spreadsheet and then transferred to an SPSS, version 27 (IBM Corporation) database. These data were validated to include only eligible patients. Eligible patients who visited the clinic during 2018 were grouped into the 2018 Usual Care Group, while eligible patients who visited the clinic during 2019 were grouped into either the 2019 Kanvas App Group or 2019 Usual Care Group based on their decision to self-select to download and register on the phone-based Kanvas app. A chi-square statistic was calculated to compare the proportions of the 3 study groups, who were classified as provider discharged or self-discharged. The remaining outcome variables, including the total number of clinic visits each patient attended, the total charges for services, and the total payments received by the clinic per patient, were addressed through a 1-way ANOVA comparing the outcome variables among the 3 study groups. Significant main effects (*P*<.05) of these ANOVA equations indicated post hoc comparisons of the group means using the Tukey least significant differences.

## Results

**Figure 4:**
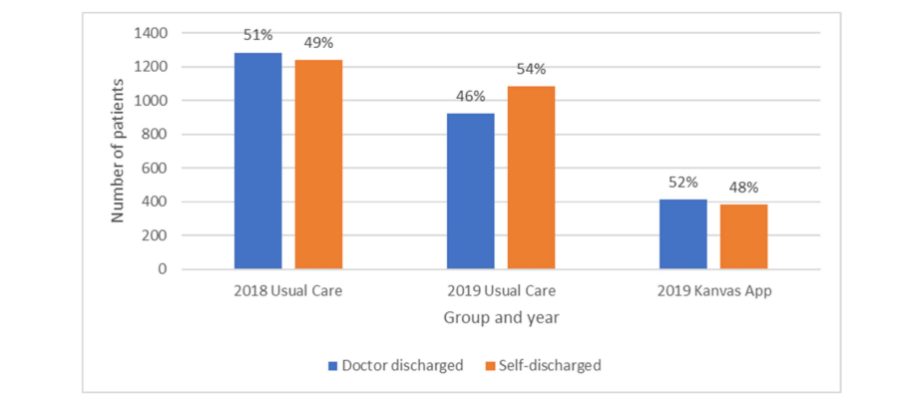
Provider vs self-discharge by year and group.

A total of 5844 patient records were reviewed, and 5328 (91.2%) were included in the analysis. Of these 5328 patients, 2523 (47.4%) were in the 2018 Usual Care Group, 2006 (37.7%) self-selected the 2019 Usual Care Group, and the remaining 799 (15%) self-selected the 2019 Kanvas App Group. Figure 4 indicates that 51% (n=1284) of the patients in the 2018 Usual Care Group were provider discharged, while the remaining 49% (n=2523) were self-discharged. Figure 4 also indicates that among the 2019 Usual Care Group, 46% (n=1084) were provider discharged and 54% (n=2007) were self-discharged. Finally, among the 2019 Kanvas App Group, 52% (n=384) were provider discharged and 48% (n=799) were self-discharged (χ^2^_2_=13.83, *P*<.001).

Table 1 presents the results of the 1-way ANOVA comparing the 3 study groups on the total number of clinic visits each patient attended, the total charges for services, and the total payments received by the clinic per patient. This analysis indicated that patients who self-selected the 2019 Kanvas App Group had significantly more total patient visits (13.21, SD 12.09; *P*<.001) when compared with the 2018 Usual Care Group (10.73, SD 9.80) and the 2019 Usual Care Group (11.35, SD 11.10). A similar pattern in the data emerged with the 2019 Kanvas App Group exhibiting significantly greater total charges for services (US $3702, SD US $3299; *P*<.001) than either the 2019 Usual Care Group (US $3096, SD US $3002) or the 2018 Usual Care Group (US $2920, SD US $1348). Additionally, post hoc analysis further revealed that the 2019 Usual Care Group exhibited significantly greater charges than the 2018 Usual Care Group. Finally, Table 1 indicates that the clinic received significantly greater total payments per patient (*P=*.02) from the 2019 Kanvas App Group (US $1513, SD US $1517) compared to the 2018 Usual Care Group (US $1348, SD US $1410), while the total payments from the 2019 Usual Care Group (US $1415, SD US $1549) was not statistically different from the other 2 study groups.

**Table 1:** Charges, payments, patient visits per patient and group^a^.

Variable	2018 Usual Care, mean (SD)	2019 Usual Care, mean (SD)	2019 Kanvas App, mean (SD)	1-Way ANOVA	
				*F* score	*P* value
Total patient visits	10.73 (9.80)^a^	11.35 (11.10)^a^	13.21 (12.09)^a^	16.48	<.001
Charges (US $)	2920.62 (1348.4)^a^	3096.18 (3002.5)^a^	3702.71 (3299.9)^a^	21.94	<.001
Payments	1348.44 (1410.9)^a^	1415.09 (1549.6)^a^	1513.62 (1517.7)^a^	3.81	.02

^a^Means with different letters for an individual variable are significantly different at *P*<.05.

## Discussion

### Principal Findings

The findings indicate that patients attending a physical health clinic who choose to adopt a phone-based app to complement their care exhibited a higher rate of provider discharge compared to patients who did not adopt the phone-base app. This greater rate of provider discharges among the patients who adopted the phone-based app likely contributed to this group also attending more clinic visits and generating more clinic charges and payments.

The findings of this study are consistent with previous studies and address a number of gaps in the literature. Previous investigators have reported that technology-based health interventions including phone apps can increase adherence with prescribed therapies [18-22]. This study is one of the first to demonstrate the efficacy of a phone app to increase adherence with prescribed physical therapy, resulting in greater revenue for the clinic. These findings may be employed to address the high rates of patients who do not complete their prescribed course of physical therapy or those who self-discharge from care [23-25].

### Strengths and Limitations

This study contains a number of limitations and strengths that may direct future inquiry into this area. The validity of this study is strengthened by the large sample size collected over multiple clinical sites and the use of the electronic medical record as the source of outcome variables. The data employed in the analysis are also clinically valid because charges for services and payments are based on the electronic medical record. Although encouraging, these findings must be interpreted cautiously due to a number of methodological limitations. First, the source of the data for this study was a retrospective review of the electronic medical record. Although a rich source of data, the electronic medical record is limited by the lack of consistency and expertise of individuals entering data into the system and the existence of missing data, which are not easily reconstructed [26]. The second limitation in this study was that patients in the 2019 study groups had the option to choose whether or not to download the Kanvas app. The decision to self-select the adoption of this mobile app may have been made by patients who were more likely to be provider discharged, attend more clinic visits, and generate more charges and payments. Future studies may wish to randomly assign patients who are initially willing to download the Kanvas app to groups who are and are not provided with the Kanvas app, to minimize the impact of this self-selection bias. Future investigators may also describe the reasons patients self-selected not to download the Kanvas app and address those reasons in future trials. The large sample examined for this study increased the external validity of the findings, although it increased the likelihood of detecting statistical significance of a small effect size.

Future clinicians will need to weigh the anticipated benefits and costs that may accompany providing patients with a phone-based app to complement their care. The costs include not only the phone-based app but also the cost of staff to monitor and interact with patients using the app. The benefits may include higher rates of adherence with prescribed therapy, as well as the return on investment of the technology, including how the technology affects revenue. Patients who self-selected the Kanvas app on average had approximately 2-3 more clinic visits with roughly US $6000-$8000 more charges and US $1000-$2000 more in payments than the groups who were not able to access the app (2018 Usual Care) or chose not to download the app (2019 Usual Care). Although numerous studies have reported the clinical efficacy of technology-based health interventions, including phone apps, few studies have consistently found these interventions generate revenue or are at least cost neutral while benefiting patients [25,26]. Finally, the validity of the findings may be limited because the individual patient’s use of the Kanvas app was not monitored. The methodology employed in this study did not monitor the type or duration of interaction the patient engaged with the app. Future studies may wish to study the time spent with the app and the type of activities engaged in with the app that contributed to increased patient adherence with prescribe physical therapy treatments.

### Conclusion

These findings support the efficacy of the Kanvas app to increase provider discharge rates and increase clinic visits, resulting in greater charges and payments among patients attending a chiropractic and rehabilitation clinic. Future investigators need to employ more rigorous methods to confirm these findings. Clinicians need to weigh the anticipated benefits of the Kanvas app against the cost and staff involvement in managing this app.

## Data Availability

The data sets generated or analyzed during this study are available from the corresponding author on reasonable request.
